# Impact of the COVID-19 pandemic on the health behaviour of university students in Hamburg, Germany: Ralf Reintjes

**DOI:** 10.1093/eurpub/ckac131.571

**Published:** 2022-10-25

**Authors:** M Loof, F Czech, M Fiedler, M Kewitz, L Kunze, Y Munusy, R Reintjes

**Affiliations:** Health Sciences, Hamburg University of Applied Sciences, Hamburg, Germany

## Abstract

**Background:**

The academic surveillance system SuSy has been collecting and analysing data regarding the health behaviour of health sciences and medical technology students twice a year since 2014. The aims of the project are to identify risk factors as well as trends and to explore the impact of the COVID-19 pandemic on university students. The data provides a suitable basis for planning and implementing health promotion and prevention measures to improve students’ health.

**Methods:**

A cross-sectional assessment as part of a rolling cohort analysis overall captured data of more than 3,000 cases using quantitative paper-pencil and online questionnaires at a German university in Hamburg. In autumn 2021, 257 students (193 women, 62 men), aged 18 to 54 years participated in the survey. However, trends are only described among undergraduate health sciences students (n ≈ 150 each survey).

**Results:**

During the COVID-19 pandemic the health behaviour of students has changed considerably in many aspects. After remaining almost constant for five years the percentage of students consuming analgetic drugs regularly increased significantly from 45 % to 68 % during the first two years of the pandemic. The percentage of students consuming soporific drugs and tranquilizers has doubled during the same time and reached a new high (28 %) in autumn 2021. Parallel to the implementation and relaxation of relevant restrictions the consumption of alcohol first decreased noticeably, but then rose to an even higher level. Also, the percentage of students exhibiting low mental well-being more than doubled from 18 % to 43 %.

**Conclusions:**

The surveys indicate that students started to engage in riskier health behaviour during the COVID-19 pandemic while being subjected to low mental well-being. In addition to the observed vulnerability, further research regarding students’ health is required to identify potential, yet generalisable health risks to enhance and initiate expedient preventive measures.

**Key messages:**

• The development, implementation and regular enhancement of a student health-related surveillance system is advisable.

• University students need to receive more public health attention in the future.

